# Explorative search of distributed bio-data to answer complex biomedical questions

**DOI:** 10.1186/1471-2105-15-S1-S3

**Published:** 2014-01-10

**Authors:** Marco Masseroli, Matteo Picozzi, Giorgio Ghisalberti, Stefano Ceri

**Affiliations:** 1 Dipartimento di Elettronica, Informazione e Bioingegneria, Politecnico di Milano, Milano, 20133, Italy

## Abstract

**Background:**

The huge amount of biomedical-molecular data increasingly produced is providing scientists with potentially valuable information. Yet, such data quantity makes difficult to find and extract those data that are most reliable and most related to the biomedical questions to be answered, which are increasingly complex and often involve many different biomedical-molecular aspects. Such questions can be addressed only by comprehensively searching and exploring different types of data, which frequently are ordered and provided by different data sources. Search Computing has been proposed for the management and integration of ranked results from heterogeneous search services. Here, we present its novel application to the explorative search of distributed biomedical-molecular data and the integration of the search results to answer complex biomedical questions.

**Results:**

A set of available bioinformatics search services has been modelled and registered in the Search Computing framework, and a Bioinformatics Search Computing application (Bio-SeCo) using such services has been created and made publicly available at http://www.bioinformatics.deib.polimi.it/bio-seco/seco/. It offers an integrated environment which eases search, exploration and ranking-aware combination of heterogeneous data provided by the available registered services, and supplies global results that can support answering complex multi-topic biomedical questions.

**Conclusions:**

By using Bio-SeCo, scientists can explore the very large and very heterogeneous biomedical-molecular data available. They can easily make different explorative search attempts, inspect obtained results, select the most appropriate, expand or refine them and move forward and backward in the construction of a global complex biomedical query on multiple distributed sources that could eventually find the most relevant results. Thus, it provides an extremely useful automated support for exploratory integrated bio search, which is fundamental for Life Science data driven knowledge discovery.

## Background

Data deluge of the post-genomic era is providing scientists with potentially valuable information, but makes difficult to find and extract from the available data those that are most reliable and most related to the biomedical questions to be answered. Moreover, such questions are increasingly complex and often simultaneously regard many heterogeneous aspects of an organism and its biomolecular entities. Several of these questions can be addressed only by searching, extracting, integrating and comprehensively querying different types of data, which are distributed in several data sources and often inherently ordered or associated with ranked confidence values. Usually, scientists manually explore these data using the individual search services available and struggle in combining intermediate results in order to find the most adequate answers to their global questions.

Several data integration platforms and workflow systems [1] have been created to query and combine available data and services from heterogeneous sources in order to explore existing information and extract new knowledge. Proposed data integration approaches can be grouped with respect to the adopted integration techniques or interaction paradigms. The former ones include information linkage, data warehousing, mediator based systems and service integration methods. Information linkage implementations, like SRS [2] or NCBI Entrez [3], enable users to interrogate several sources through a single Web site and provide results with links to the data sources; yet, they do not integrate the retrieved data. Fully materialized systems, like EnsMart [4] or BioWarehouse [5], integrate data within a warehouse according to a local schema. This approach allows performing easily complex computations on the integrated data, but requires updating often the data warehouse, which generally is a complex task. Mediator based systems, like TAMBIS [6] or BioMart [7], are designed to query remotely distributed sources through a virtual mediated schema; the query on the mediated schema is transformed in queries over the schemata of the diverse sources and the retrieved data are processed locally. In mediated approaches data remain in the original sources without being materialized locally; thus, mediator based approaches provide up-to-date data, but complex computations on the data are a challenging task. Service integration approaches require registering the services in order to describe them according to an integration model. Among others, Mork et al. [8] proposed an entity-based model to integrate data from diverse services; they suggested to register services through a DSL (Domain Specific Language), based on an eXtensible Markup Language (XML) file, and map them onto the entities described in the model.

Among interaction paradigms, the path-based approach is similar to the exploratory one used in our work; it is founded on a semantic graph, built according to links available between sources, which enables users to compose queries by selecting entities from the graph. Biozon [9], GenoQuery [10] and the BioGuide (http://www.bioguide-project.net/) tool family (e.g. BioGuideSRS [11]) are examples of such approach implementations. Several other types of query interfaces have also been proposed. Recently, Latendresse and Karp [12] presented their Structured Advanced Query Page as an original interface to query a unique integrated database containing multiple data types.

Notable examples of workflow systems supporting service and data integration include Taverna [13], Wings/Pegasus [14,15], Galaxy [16], Triana [17] and Kepler [18]. Yet, Taverna, the most known and used in bioinformatics, and the other available workflow systems do not rely on a general model of the services to be integrated. Furthermore, available data integration platforms and workflow systems do not take into account, in the integration process, often available partial rankings of the data to be integrated. Thus, they cannot provide support for ranking-aware multi-topic searches. Both these limitations are addressed and overcome by Search Computing (http://www.search-computing.org/). It has been proposed as a new software framework that provides the abstractions, foundations, methods, and tools required to answer complex multi-topic queries over multiple data sources, also ranked [19]. It reaches this goal by interacting with a collection of cooperating search services and using ranking and joining of results as the dominant factors for service composition. The diverse services are described, according to a general and flexible service model, at three different levels of abstraction, i.e. at conceptual, logical and physical level [19,20]; then, they are wrapped, registered in the system and mapped onto the virtual mediated schema, which is built based on the semantic relationships between services described at service registration. These aspects originally differentiate Search Computing from previous proposals for service registration and integration of data from diverse services, such as the one from Mork et al. [8].

Here, we illustrate and discuss our novel work to support explorative integrated bio search and ranking-aware combination of distributed biomedical-molecular data, aimed at answering multi-topic complex biomedical questions. This work complements a previous study [21] of the envisaged relevance of Search Computing to the Life Sciences, in particular to information integration and support for Life Sciences ordered data. The foundation of the extension of Search Computing in support of explorative searches in the complex biomedical-molecular scenarios was shortly introduced in [22] and [23]; here such extension is thoroughly illustrated and discussed, focusing on a paradigmatic bioinformatics use case. By supporting interactive explorative multi-topic data searches, the work here presented significantly extends a previous approach [24] focused only on the efficient execution of predefined single global multi-topic queries over multiple ranked search services. The demonstrator prototype initially developed to implement such previous approach [25] is significantly extended and enhanced by the original Web application here presented and made publicly available. Besides allowing querying diverse services and integrating their provided data on-the-fly, it additionally supports exploration (inspection and selection) of intermediate partial results, as well as their expansion and refinement through search query modification and extension. Furthermore, it enables users to attribute different weights to results from diverse sources.

## Results

**Figure 1:**
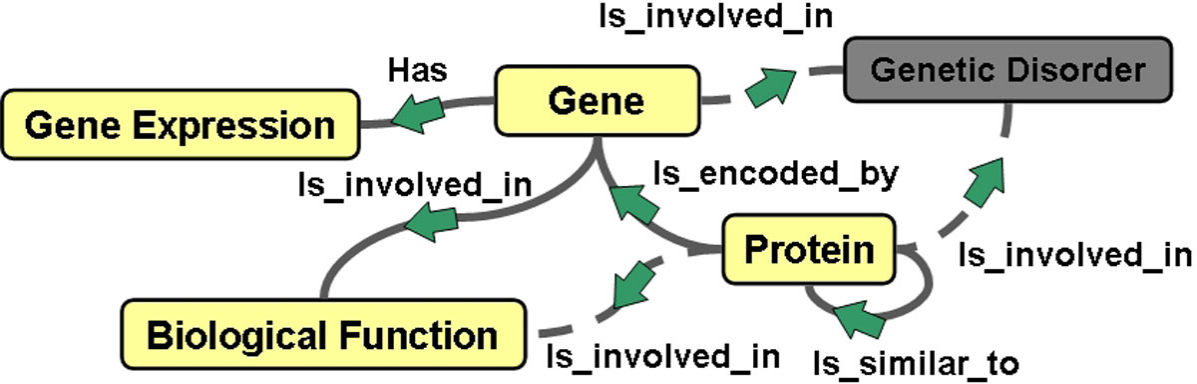
**The biomedical-molecular Semantic Resource Framework created in Bio-SeCo**. Boxes represent the topics of the search services registered in the Bio-SeCo framework; lines represent the semantic connections defined at service registration time between the registered services. Light yellow boxes and full line arches show an example of exploration and query of the biomedical-molecular Semantic Resource Framework.

We modelled and registered in the Search Computing framework, as described in the *Methods *section, a set of bioinformatics services and their semantic connections, thus creating the Semantic Resource Framework shown in Figure 1. Leveraging it, we created a Bioinformatics Search Computing application (Bio-SeCo) (http://www.search-computing.org/UIDemoBio/) and made it publicly accessible through a Web interface at http://www.bioinformatics.deib.polimi.it/bio-seco/seco/. It enables explorative search and automatic ranking-aware integration of bio-data provided by the individual services registered in the framework. In the Bio-SeCo user interface, the registered services can be used and combined, according to their connection patterns defined at service registration time, to explore and globally search the data that they provide. Initial individual search results, obtained by setting search input parameters, can be combined, taking into account individual rankings, in order to refine or expand initial searches. In so doing, scientists can easily use the registered services to find, in an explorative way, answers to complex multi-topic biomedical-molecular questions such as "*Which are the biological functions of the genes known to be significantly over expressed in the anatomical organ × and to have mutations associated with the genetic disorder Y?*", or "*Which are the proteins most likely homolog of a given protein × that are involved in biological function Y and encoded by genes down expressed in the biological condition Z?*".

**Figure 2:**
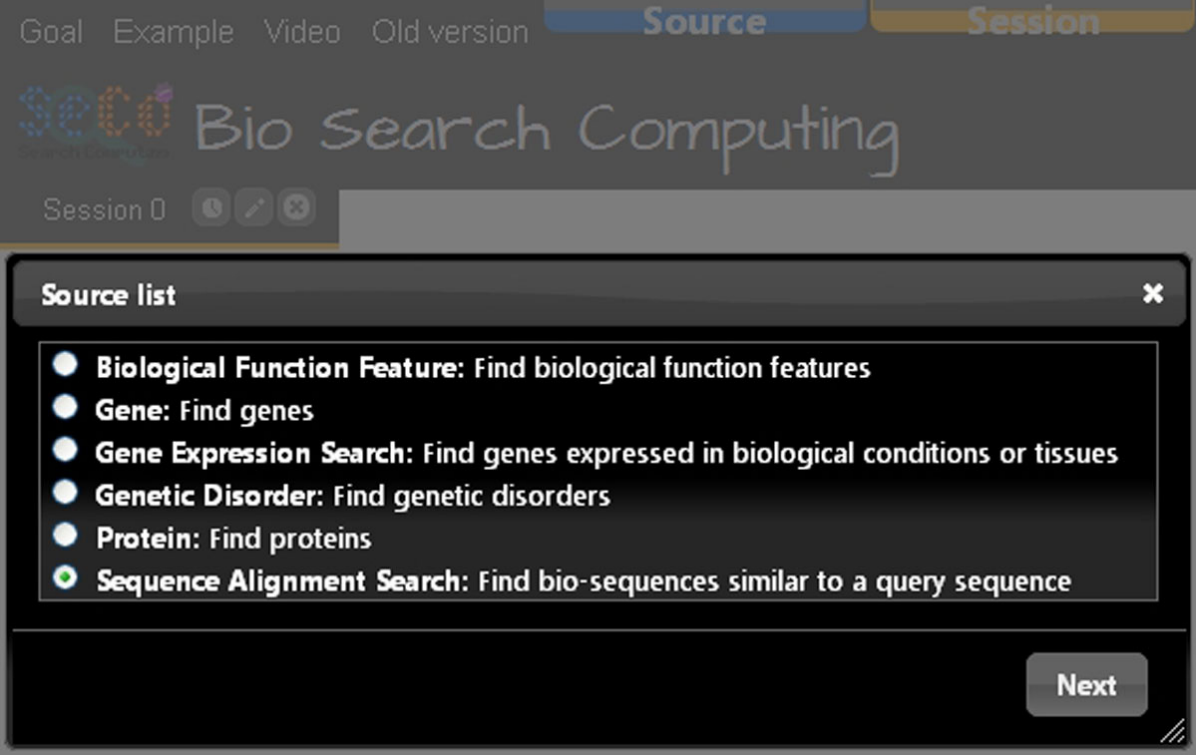
**Screenshot of the initial menu of the Bio-SeCo user interface**. The list of topics covered by the services registered in Bio-SeCo for search computing is shown.

**Figure 3:**
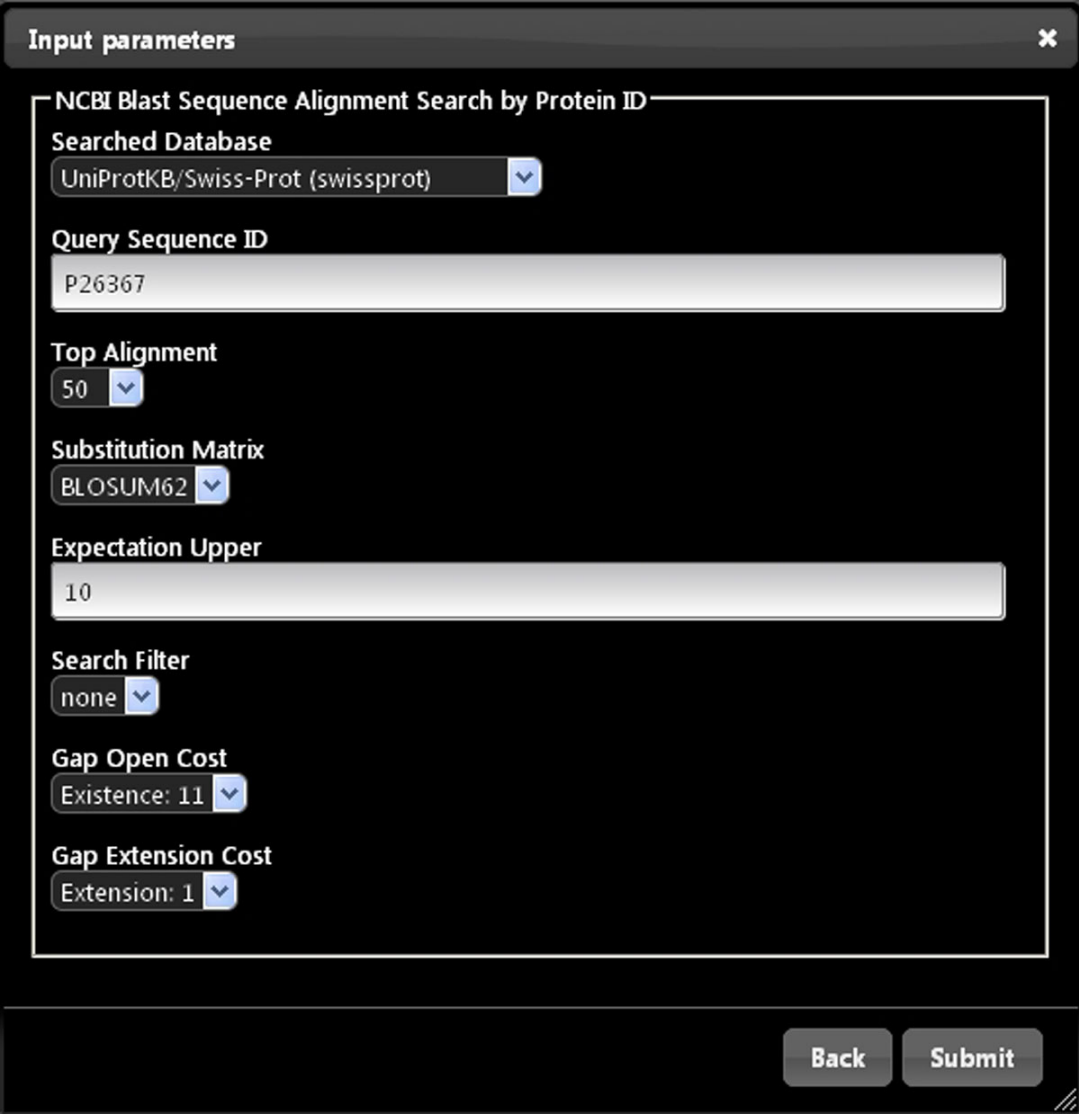
**User interface to set input parameters of the "NCBI Blast Sequence Alignment Search" service registered in Bio-SeCo**. Input values to search, with default BLAST parameter values, for proteins in the UniProtKB/Swiss-Prot database similar to the protein with http://www.uniprot.org/uniprot/P26367 ID are shown as an example.

As a use case example of Bio-SeCo, let us suppose a scientist wants to explore available data regarding genes and proteins in order to find which are the genes (if they exist) that encode proteins in different organisms with high sequence similarity to an amino acid sequence *X *and have some biomedical features in common (e.g. they are significantly co-expressed in the same biological tissue or condition *Y *and involved in a biological process *Z*). Using the resources registered in Bio-SeCo (Figure 2), for example, such scientist can first run a sequence alignment search (e.g. using the NCBI Blast service with default BLAST parameters), in order to look for proteins similar to an amino acid sequence *X *(e.g. the protein with http://www.uniprot.org/uniprot/P26367) in a selected amino acid sequence database (e.g. *UniProtKB/Swiss-Prot*). Figure 3 illustrates the Bio-SeCo interface where the user can specify the input parameters for such a search.

**Figure 4:**
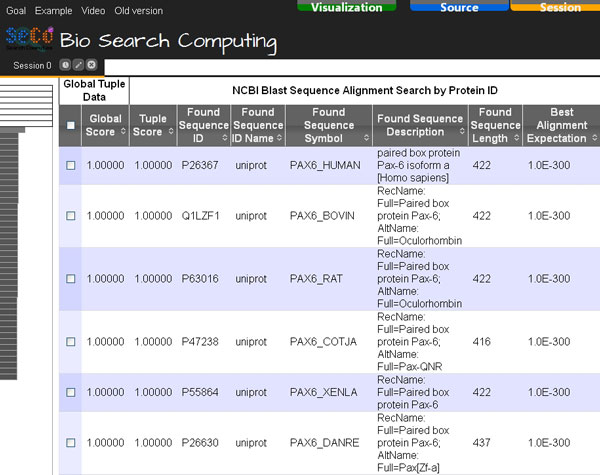
**Bio-SeCo result Table View**. The first results of the "NCBI Blast Sequence Alignment Search" for the query protein with http://www.uniprot.org/uniprot/P26367 ID are shown.

**Figure 5:**
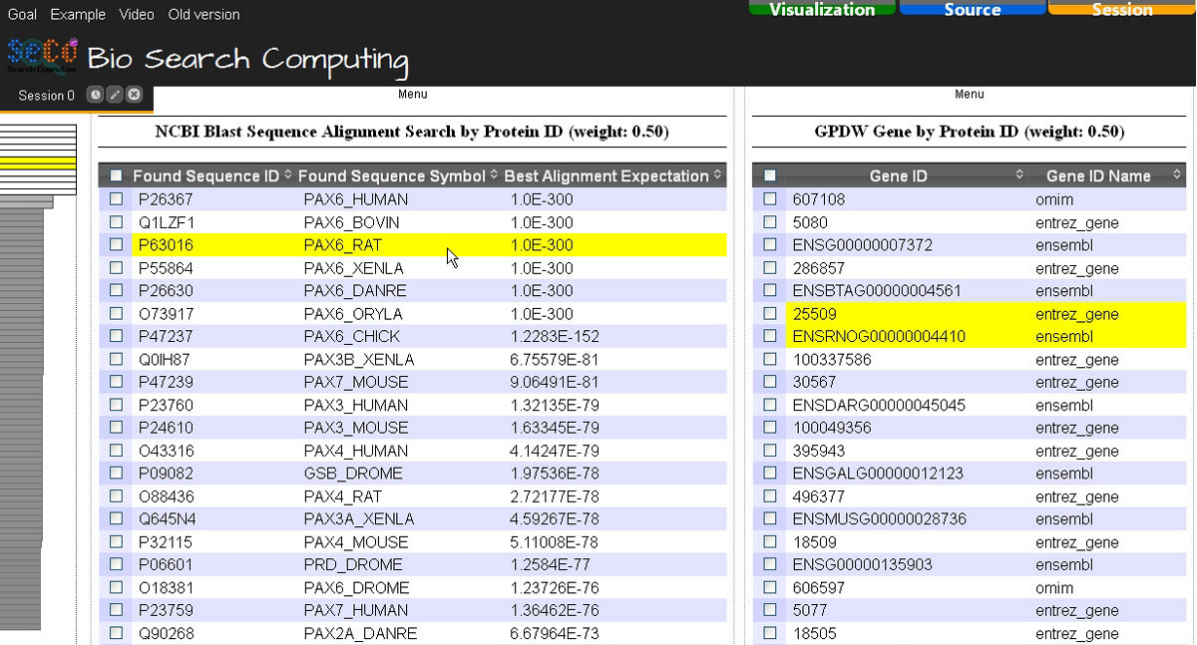
**Bio-SeCo result Atom View**. The joined search results of the "NCBI Blast Sequence Alignment Search" and "GPDW Protein coding Gene" services for the query protein with http://www.uniprot.org/uniprot/P26367 ID are shown. Pointing on a data record found by a service, also the related data record(s) found by the other service(s) involved in the multi-topic search is(are) highlighted together with the position(s) of the data record in the global ranking (shown on the left) of the search results. Single service relative weights used are shown close to the name of the service to which each of them refers.

Then, the scientist can explore the obtained search results (Figure 4), select the most similar proteins found or some of them (e.g. the ten most similar ones or only those of some selected organisms) and automatically retrieve the codifying gene of each of them by using the GPDW Protein coding Gene query service, which is registered in Bio-SeCo as connected to the NCBI Blast service. Figure 5 shows the Atom View of the obtained results. Such atomic data view implemented in the Bio-SeCo user interface is particularly useful to synthetically display the distinct values found for the relevant attributes of each service involved in the performed multi-topic search. By moving the mouse pointer on a data record found by a service, also the related data record(s) found by the other service(s) involved in the multi-topic search are highlighted. Furthermore, also the position(s) of the data record in the global ranking of the search results (automatically computed according to the ranking of the partial search results provided by each of the involved services) is(are) highlighted (shown on the left in Figure 5).

**Figure 6:**
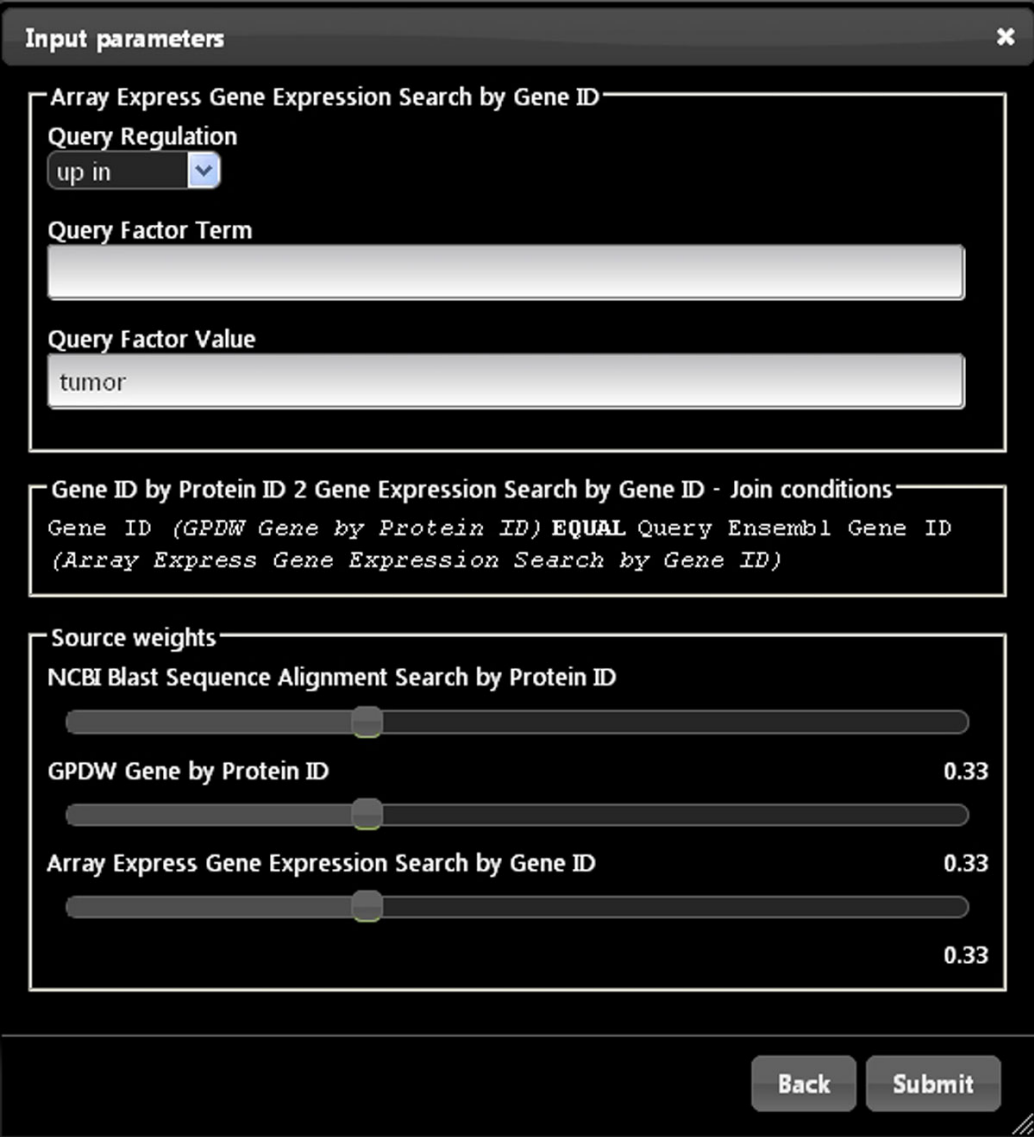
**User interface to set additional input parameters to refine obtained search results and to interactively change single service relative weights**. The interface for the "Array Express Gene Expression Search" service registered in Bio-SeCo is shown as an example with input parameter values to expand the search for genes with expression significantly *up *regulated in *tumor *using equal single service relative weights.

Next, the scientist can search for biomedical features shared among the retrieved genes. For instance, by using the Array Express gene expression search and GPDW Gene Biological Function Feature annotation services, he/she can explore if some of the initially found genes are known to be significantly co-expressed (e.g. *up *regulated) in the same biological tissue or condition *Y *(e.g. in *tumor*) and involved in a biological process Z (e.g. in *regulation of apoptotic process*). Figure 6 shows the Bio-SeCo interface where the user can set the additional input parameters to refine the obtained search results by using the Array Express gene expression search service, which is registered in Bio-SeCo as connected to the GPDW Protein coding Gene query service. Using the same interface, the user can also set the single service relative weights used to compute the result global scores.

By performing the exploratory search steps of the use case example above described, the scientist can explore the biomedical-molecular Semantic Resource Framework defined by the bioinformatics services registered in Bio-SeCo (Figure 1). In so doing, he/she can compose and submit a global query that might find the answer to his/her original complex multi-topic question: "*Which genes encode proteins in different organisms with high sequence similarity to a given protein X, are significantly over co-expressed in the same given biological tissue or condition Y and are involved in the biological process Z?*" The possibility to easily construct in an explorative way such complex biomedical queries and run them efficiently across multiple distributed sources allows global evaluations of available bio-data that can unveil unexpected results and lead to new biomedical knowledge discoveries. On December 18^th ^2013, we run the above example global query by using equal service relative weights and setting input parameter values with the human *Paired box protein Pax-6 isoform a *protein [UniProt:*P26367*] ID as amino acid sequence *X*, *tumor *as pathological biological condition *Y*, and *regulation of apoptotic process *as biological process *Z*.

**Figure 7:**
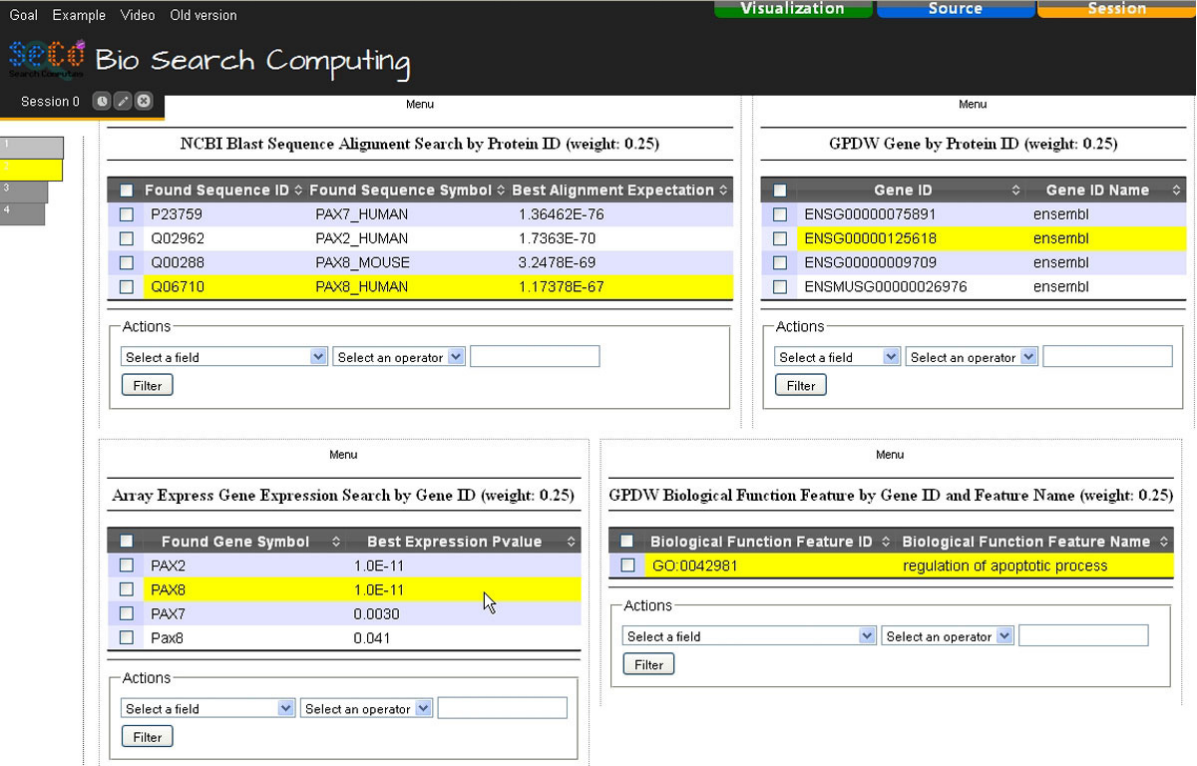
**Bio-SeCo result Atom View**. Global results obtained from the initial search, for query protein with http://www.uniprot.org/uniprot/P26367 ID by using "NCBI Blast Sequence Alignment Search" and "GPDW Protein coding Gene" services, refined by searching with "Array Express Gene Expression Search" and "GPDW Gene Biological Function Feature annotation" services for initially found genes significantly *over *co-expressed in *tumor *and involved in the biological process of *regulation of apoptotic process*, using equal single service relative weights. Pointing on a data record found by a service, also the related data record(s) found by the other service(s) involved in the multi-topic search is(are) highlighted together with the position(s) of the data record in the global ranking (shown on the left) of the search results.

Unpredictably, in the bio-data then available we found the human *PAX2*, *PAX8 *and *PAX7 *and mouse *Pax8 *genes, ordered by their global scores of 0.80813, 0.80578, 0.62056 and 0.58860, respectively (with 1.0 as best score) (Figure 7). These scores take into account both partial rankings induced by the sequence similarity expectations and gene expression *p*-values, which both have dimensionless values in the same [0.0 - 1.0] interval, provided by the NCBI Blast and Array Express services called in the global query. The four genes found encode, respectively, the human *Paired box protein Pax-2*, human *Paired box protein Pax-8*, human *Paired box protein Pax-7 *and mouse *Paired box protein Pax-8*. These proteins respectively have 1.73781 E^-70^, 1.17479 E^-67^, 1.3658 E^-76 ^and 3.2506 E^-69 ^expectation of sequence similarity to the input human *Paired box protein Pax-6 isoform a *protein. Their encoding genes are all significantly *over *expressed in *tumor *with 1.0 E^-11^, 1.0 E^-11^, 0.0030 and 0.041 *p*-value, respectively, and all of them are involved in *regulation of apoptotic process*. Notice that the human *PAX6 *gene, which encodes the input human *Paired box protein Pax-6 isoform a *protein, is not among the found genes since it is not known to be involved in *regulation of apoptotic process*. Furthermore, although the human *PAX7 *gene encodes a protein much more similar to the input protein than the proteins encoded by the other genes found, it is not in the top position of the ordered global results found since it is less significantly over expressed in *tumor *than the human *PAX2 *and *PAX8 *genes. This result has been found very quickly thanks to the support provided by Bio-SeCo; to our knowledge currently no other computational systems are able to provide it. Furthermore, the explorative search peculiarities of Bio-SeCo enable the user to easily look at the intermediate partial findings that led to discover the final global result, i.e. its supporting evidence based on the available data.

## Discussion

The created Bio-SeCo application implements a novel exploratory search interaction paradigm and supports the user in performing a progressive step-by-step construction of the search query by exploring the data provided by the available services registered in Bio-SeCo. This aspect of expanding an initial query - according to the liquid query paradigm [26] - after evaluating its provided results, in order to refine or extend them, innovatively differentiates our exploration approach from the path-based one.

Conversely, both approaches use a graph of sources to express the queries; thus, Figure 1 could be obtained also in path-based systems [9-11]. Usually scientists perform manually such supervised exploration of data by using the individual tools available, save somewhere (e.g. within a spread sheet) single search results and manually combine/compare them in order to identify common patterns and try to find answer to their global questions. Bio-SeCo offers an integrated environment where to perform such data exploration, which automatically saves intermediate results, combines them taking into account their partial order and supplies ordered global results. Furthermore, Bio-SeCo offers multiple alternative and interchangeable types of result visualization, i.e. table, atom and scatter plot views, with also the possibility to easily integrate new advanced visualizations.

The order of the provided results is induced by their global scores, computed on the basis of the Fagin's method [27] and according to a score function defined as combination of partial scores of intermediate ranked results, as described in the *Methods *section. This choice seams to be the most appropriate for Bio-SeCo, which aims at quickly giving global ordered answer sets to user complex searches on multiple combined search services that provide individual rankings, possibly incomplete and with ties. It was positively evaluated by the users who provided feedbacks about the relevance of the system and its ranking strategy. An alternative to Fagin's method could be the very promising BioConsert method, recently presented by Cohen-Boulakia et al. [28]. They proposed to rank answer sets, retrieved for a user query, according to a median-based consensus ranking generated on the basis of the results of a set of ranking methods and reflecting their common points. Since finding a median of rankings with ties is a NP-hard problem, they proposed an interesting heuristic to generate such a consensus ranking. It performs well with the datasets considered in [28]; yet, being a greedy heuristic, unfortunately it is not guaranteed to always perform as well for all data sets.

**Figure 8:**
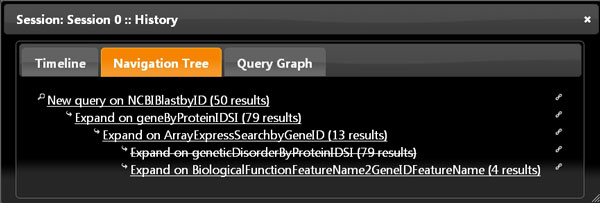
**Example of History Navigation Tree of a bio-data exploration through search services registered in Bio-SeCo**. From an initial query (using the NCBI Blast service), three subsequent query expansion refinements (using the GPDW Protein coding Gene, Array Express gene expression search and GPDW Protein Genetic Disorder services, respectively); then one backward step (to the query expansion refinement using the Array Express gene expression search service, by undoing the query expansion refinement performed using the GPDW Protein Genetic Disorder service) and a following query expansion (using the GPDW Gene Biological Function Feature annotation service).

Our work here reported enhances and significantly extends an initial demonstrator prototype previously developed, which only supported predefined global multi-topic queries over ranked search services [25]. Beside modelling and registering in Bio-SeCo additional new services, in our novel work we created a user-friendly interactive Web interface that offers public access to Bio-SeCo at http://www.bioinformatics.deib.polimi.it/bio-seco/seco/ and supports explorative multi-topic bio-data searches. It enables the user to explore the very large and very heterogeneous biomedical-molecular data available, allowing he/she to easily expand or refine a previous query, make different attempts, inspect obtained results through topic-driven visualizations and move forward and backward in an activity that would eventually find the most relevant results, in case after several unsuccessful attempts (Figure 8). In so doing, the user interactively constructs a multi-topic global query, by defining query elements and constrains for each considered topic during the exploration of the available bio-data. Such global query is then executed in the Bio-SeCo environment, where it is optimized according to the expected invocation costs (based on expected intermediate and final result sizes) of each individual service used to answer the global query.

The future development of Bio-SeCo will focus on further extending its Semantic Resource Framework by registering in Bio-SeCo additional bioinformatics services, thus supporting a wider variety of biomedical questions, even more complex. It will also include the aspect of guiding user exploration of available resources towards the ones that provide more appropriate data according to the user preferences and strategies. To this regard, path-based systems like Biozon [9] and BioGuideSRS [11] are important reference for systems aimed at assisting scientists in searching for relevant data within external sources while taking their predilections and policies into account.

## Conclusions

By using available services to search biomedical-molecular data and taking advantage of the ranking attributes that they define, the here described Bioinformatics Search Computing application allows efficient exploration of available bio-data and search for globally ranked answers to complex multi-topic biomedical questions. In so doing, it offers a valuable and powerful automated support for exploratory integrated bio searches at the basis of Life Science data driven knowledge discovery.

## Methods

### Search Computing framework

**Figure 9:**
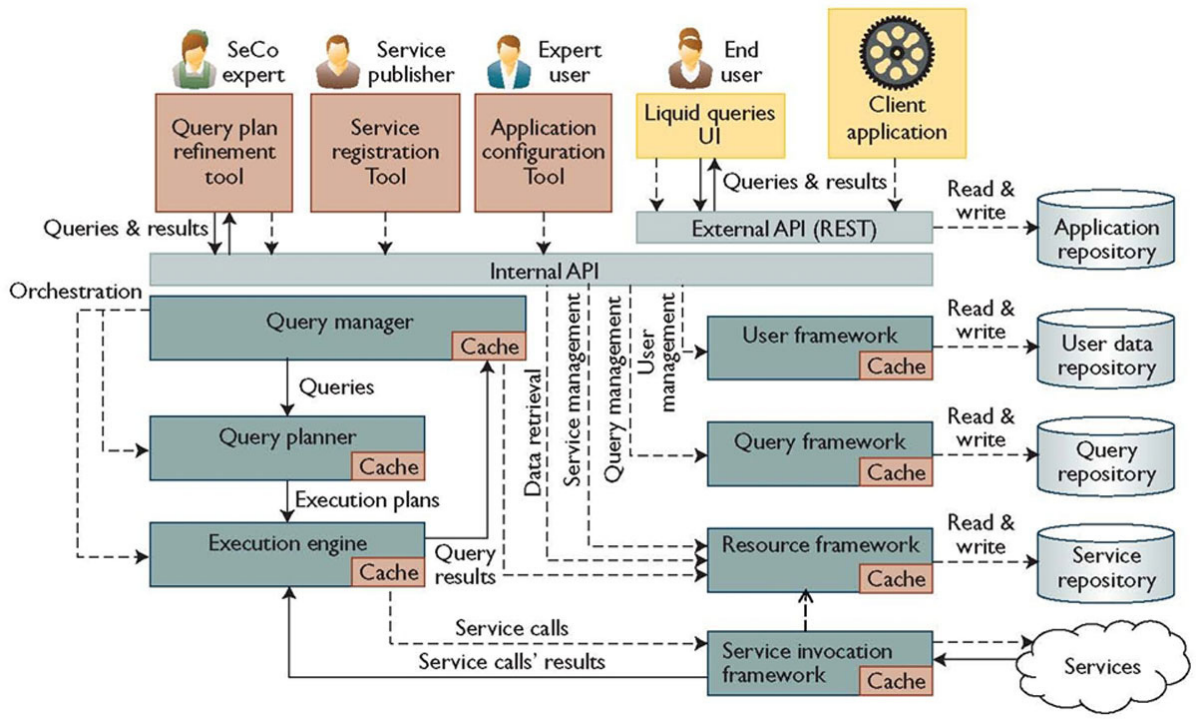
**Search Computing architecture**. The multiple different components of the architecture are shown together with the operation flow interconnecting them.

To compute answers to complex multi-topic queries over multiple data sources, also ranked, we used the Search Computing software framework (http://www.search-computing.org/) [19]. It allows interacting with a collection of cooperating search services and orchestrating them by using ranking and joining of results as the main factors for service composition. It covers both server-side (service modelling, workflow management, query planning and execution, data materialization, etc.) and client-side (user interaction, service registration, data visualization, etc.) aspects. Towards this aim, the Search Computing framework includes a variety of tools covering service development and publishing, query execution, as well as application registration and query tuning. Figure 9 presents the overall conceptual architecture of the framework. A service registration environment eases the creation of wrappers to adapt existing services to the Search Computing framework. A repository stores the definitions of wrappers and registered data sources, which are used for the deployment of specific search-based applications. A client-side user interface component enables end users to submit queries and visualize results. It allows several views over composed data, ranging from tabular to atomic, and supports user-centred operations to explore the search data space.

**Figure 10:**
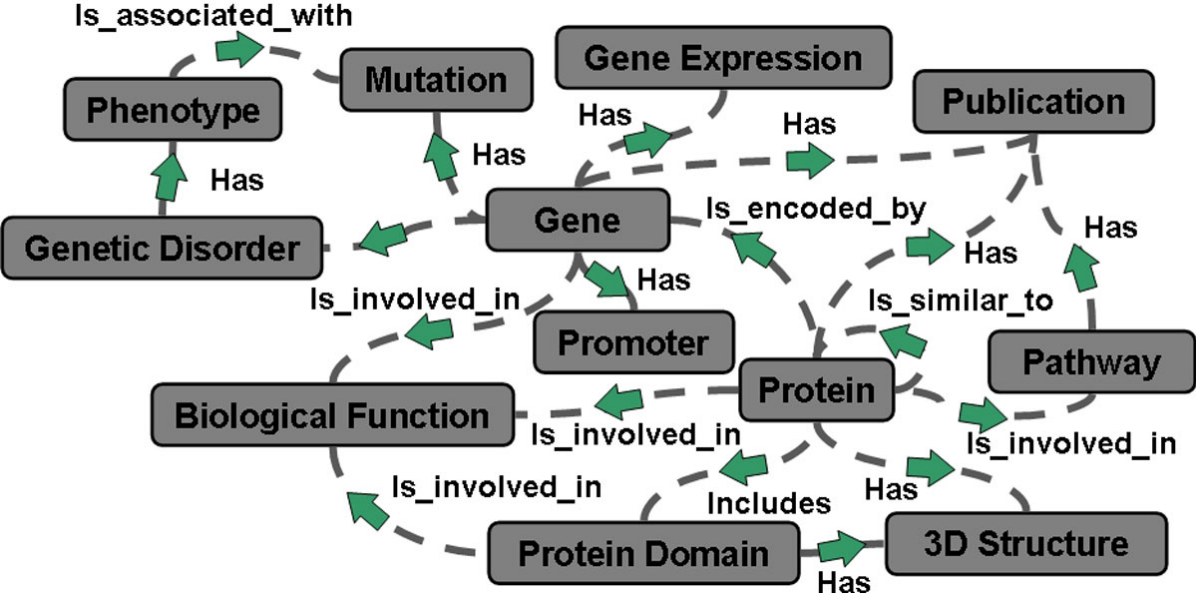
**Example of biomedical-molecular Semantic Resource Framework defined through search service registration**. Boxes represent the topics of the search services registered in the framework; lines represent the semantic connections defined at service registration time between the registered services.

In order to support answering complex multi-topic queries over distributed data sources also ranked, Search Computing provides a platform which allows expressing requests over multiple search services registered in the Search Computing framework, such that the global results of the integrated requests take account of the rankings of individual search results. This is enabled by the way in which, at service registration time, the search services - and their relationships - to be used for search computing are conceptually, logically and physically described in the framework according to the *service mart *model [20]. Briefly, this service description consists of (i) a *service mart*, which defines the type of resource that the service provides, (ii) the service associated *access patterns *with their input, output and output ranked attributes (if the service produces results ordered on the values of these attributes), and (iii) the specific *service interface *implementation used to call the service. It also includes the binding between the service associated service mart and the operations to be invoked on the service, with their input, output and output ranked attributes, as described by the used service associated access patterns. Thus, this description defines the nodes of a resource framework and how obtaining the type of data that such nodes represent by using the available services registered in the Search Computing framework. Pair-wise coupling of service marts is also defined at service registration time through *connection patterns*, which define resource framework links and specify service connection semantics. Such Semantic Resource Framework [29] is the basis of the Search Computing information exploration paradigm. Figure 10 depicts an example of Semantic Resource Framework covering several biomedical-molecular topics and their relationships, which can be created by registering some of the numerous bioinformatics services available [30] in the Search Computing framework. Such resource framework can then be leveraged for computing explorative multi-topic biomedical searches.

### Bioinformatics service modelling, registration and querying for search computing

In order to create our Bio-SeCo application, we first selected a set of typical biomedical-molecular topics (i.e. Protein, Gene, Gene Expression, Biological Function and Genetic Disorder) to be included in Bio-SeCo. According to the *service mart *modelling approach [20], we modelled the service marts (i.e. the generalized and normalized conceptual descriptions) of bioinformatics services that provide data about such topics. We did so by identifying their main and common attributes and normalizing their names. We also defined the semantic *connection patterns*, i.e. the pair-wise coupling, between service marts of services that provide data about different topics. This was done by identifying pairs of normalized attributes of the connected service marts and defining their comparison predicates, as conjunctive Boolean expressions, that allow joining their values semantically.

Then, using available Search Computing tools, we registered in Bio-SeCo some bioinformatics search services that provide data about the selected biomedical-molecular topics and their semantic associations. They include two BLAST sequence alignment and search services available at Washington University (WU) [31] and National Center for Biotechnology Information (NCBI) [32], respectively, the search engine over the Array Express repository of gene expression data [33], and five query services over our Genomic and Proteomic Data Warehouse (GPDW) publicly available at http://www.bioinformatics.deib.polimi.it/GPKB/[34]. The latter ones provide access to Gene, Protein and their Genetic Disorder and Biological Function Feature (i.e. Gene Ontology Biological Process, Molecular Function and Cellular Component) annotation data.

For each service, the service registration consists in first creating a wrapper, i.e. an adapter that matches the service attributes to their normalized version defined in a modelled *service mart*, and associating the wrapper with such a service mart. Since each type of service is modelled by a single service mart, more registered services can share the same service mart, such as the two registered BLAST services. Then, one or more *access patterns *and a *service interface *are defined for each service. The latter one maps an access pattern to the wrapper of the end point of the service data source, which is used to call the service. Whereas the former ones, which can be shared by more services associated with the same service mart, are specific signatures of a service mart, with the characterization of each attribute as input (I) or output (O), depending on the role that the attribute plays in the service call. Furthermore, an output attribute can be characterized as ranked (R), if the service produces its results in an order that depends on the values of that attribute. Based on the semantic type of access pattern input and output attributes of two registered services, specific *connection patterns *between individual services are then automatically derived from the connection patterns defined at conceptual level between the service marts associated with the registered services. All these tasks can be done quite easily by following the documentation provided by the Search Computing project. As an example, the access patterns that we created to model the NCBI Blast sequence alignment search by Protein ID and GPDW Biological Function Feature by Protein ID services, together with their pair-wise coupling connection pattern, are here reported as follows.

NCBI-BLAST(SearchedDB^I^, QueryUniprotProteinID^I^, TopAlignment^I^, SubstitutionMatrix^I^, ExpectationUpper^I^, SearchFilter^I^, GapOpenCost^I^, GapExtensionCost^I^, FoundSequenceID^O^, FoundSequenceIDName^O^, FoundSequenceSymbol^O^, FoundSequenceDescription^O^, FoundSequenceLength^O^, BestAlignmentExpectation^R^)

GPDW_BiologicalFunctionFeature(ProteinID^I^, ProteinIDName^I^, BiologicalFunctionFeatureName^I^, BiologicalFunctionFeatureID^O^, BiologicalFunctionFeatureIDName^O^, BiologicalFunctionFeatureName^O^, BiologicalFunctionFeatureDefinition^O^)

ExistsProteinBiologicalFunctionFeature(NCBI-BLAST, GPDW_BiologicalFunctionFeature): [(NCBI-BLAST.FoundSequenceID = GPDW_BiologicalFunctionFeature.ProteinID) AND [(NCBI-BLAST.FoundSequenceIDName = GPDW_BiologicalFunctionFeature.ProteinIDName)]

By doing all the described service registration steps, we created the Semantic Resource Framework depicted in Figure 1. It constitutes the reference used by Bio-SeCo to enable the query, exploration and integration of the data provided by the services registered in the framework.

A query on a single search service registered in the framework is expressed based on the user inputs and service access pattern selected. Expansion of a search service query on another search service is performed, according to the liquid query paradigm [26], by composing single search service sub-queries based on their connection pattern chosen. This last specifies the output values of the first service to be used as input values to the second service, as well as their conjunctive logical conditions to be implemented in the query execution plan. In this way, an exploratory expanded query, expressed on the biomedical-molecular semantic resource network created at service registration time, can be actually formalized in concrete sub-queries posed to the search services associated with the network nodes and related each others as defined by the network arches. For example, according to the above defined *NCBI-BLAST *and *GPDW_BiologicalFunctionFeature *access patterns and their coupling connection pattern, the expansion on the network Biological Function node (i.e. *GPDW_BiologicalFunctionFeature *service) of an initial query for Protein similarity (i.e. using *NCBI-BLAST *service) is expressed through the two following sub-queries:

NCBI-BLAST(SearchedDB, QueryUniprotProteinID, TopAlignment, SubstitutionMatrix, ExpectationUpper, SearchFilter, GapOpenCost, GapExtensionCost)

GPDW_BiologicalFunctionFeature(NCBI-BLAST.FoundSequenceID, NCBI-BLAST.FoundSequenceIDName, BiologicalFunctionFeatureName)

Their execution plan provides as expanded results only those items from the first and the second sub-query that together satisfy the conjunctive logical conditions defined in the used connection pattern. Notice that join conditions used in an expanded query are clearly shown in the Bio-SeCo user interface (Figure 6). In the considered example, the expanded results include only those user selected proteins that, according to the *NCBI-BLAST *service, are similar in sequence to a user specified protein and have the user specified biological function(s), according to the *GPDW_BiologicalFunctionFeature *service. Thus, multi-service expanded results always include only the items in common in the partial results from each of the sub-queries composed, i.e. from each combined search service.

### Partial ranking composition and global scoring function

To compose individual search results of a multi-topic query, taking into account their partial rankings and provide a global score, Bio-SeCo uses a highly efficient algorithm for rank aggregation [35-37]. It takes into account the following four major aspects of the Bio-SeCo application scenario. First, individual search results are provided by single search services that are individually called and composed within Bio-SeCo; time and completeness of their answers is not guarantied. Second, ordered search results are usually partially ranked, i.e. they can include ties. Third, depending on the user chosen parameters, individual search services may provide only top k ordered results. Fourth, as specified in the previous *Methods *subsection, global ranking is defined for subsets of equal number of common partial results from each sub-query (i.e. from each single search service). Thus, consensus ranking methods, which usually exploit the fact that the same data item is found in several rankings to construct the consensus, can be straightforwardly applied to get a global ranking for the global results on the basis of their partial rankings. Based on a consensus method previously proposed by Fagin et al. [27], the ranking algorithm implemented in Bio-SeCo can efficiently compute the elements of a near-optimal aggregation of multiple partial rankings induced by a global score. This score is computed according to a scoring function defined as the weighted summation of multiple partial scores of intermediate ranked results. The scores of the individual search results, i.e. the inputs of the scoring function, are provided by the ranked attribute of every search service called in the multi-topic (i.e. multi-service) query, where the ranked attribute of each service is identified by the specific access pattern used in the query for that service. The weights of the scoring function are defined, for each registered service, as the product of a service specific and a service relative weight. The former ones are set according to the values of the ranked attribute of the specific service to which each of them refers, in order to normalize the partial rankings of each individual search to be composed in the global search. The latter ones ensure that the composed global score is in the [0.0 - 1.0] range, with 1.0 as the best score. Constrained to satisfy such global score range, through the Bio-SeCo interface the user can interactively change the default equal values of the single service relative weights (Figure 6) to attribute more/less weight, in the global ranking, to results from some of the composed search services.

## List of abbreviations used

DSL: Domain Specific Language; Bio-SeCo: Bioinformatics Search Computing application; BLAST: Basic Local Alignment Search Tool; GPDW: Genomic and Proteomic Data Warehouse; NCBI: National Center for Biotechnology Information; SRS: Sequence Retrieval System; WU: Washington University; XML: eXtensible Markup Language.

## Competing interests

The authors declare that they have no competing interests.

## Authors' contributions

MM conceived the Bio-SeCo application project here described, was responsible for its supervision, modelled the bioinformatics services for their use in search computing and wrote this manuscript.

MP developed and tested the Bio-SeCo user interface and contributed to write this manuscript.

GG implemented and tested the Bio-SeCo application, registered the modelled bioinformatics services in the Semantic Resource framework and contributed to obtain the here illustrated results.

SC contributed to the project and conceived the Search Computing explorative approach and framework.

## Declarations

This work has been partially supported by the "Search Computing" project (2008-2013), funded by the European Research Council (ERC), under the 2008 call for "IDEAS Advanced Grants".

This article has been published as part of *BMC Bioinformatics *Volume 15 Supplement 1, 2014: Integrated Bio-Search: Selected Works from the 12th International Workshop on Network Tools and Applications in Biology (NETTAB 2012). The full contents of the supplement are available online at http://www.biomedcentral.com/bmcbioinformatics/supplements/15/S1.
